# Classification of Kiwifruit Grades Based on Fruit Shape Using a Single Camera

**DOI:** 10.3390/s16071012

**Published:** 2016-06-30

**Authors:** Longsheng Fu, Shipeng Sun, Rui Li, Shaojin Wang

**Affiliations:** 1 College of Mechanical and Electronic Engineering, Northwest A&F University, Yangling 712100, China; fulsh@nwsuaf.edu.cn (L.F.); cpengfls@gmail.com (S.S.); ruili1216@nwsuaf.edu.cn (R.L.); 2 Department of Biological Systems Engineering, Washington State University, Pullman, WA 99164-6120, USA

**Keywords:** kiwifruit grading, international grading standards, Chinese grading standards, fruit shape, image processing method

## Abstract

This study aims to demonstrate the feasibility for classifying kiwifruit into shape grades by adding a single camera to current Chinese sorting lines equipped with weight sensors. Image processing methods are employed to calculate fruit length, maximum diameter of the equatorial section, and projected area. A stepwise multiple linear regression method is applied to select significant variables for predicting minimum diameter of the equatorial section and volume and to establish corresponding estimation models. Results show that length, maximum diameter of the equatorial section and weight are selected to predict the minimum diameter of the equatorial section, with the coefficient of determination of only 0.82 when compared to manual measurements. Weight and length are then selected to estimate the volume, which is in good agreement with the measured one with the coefficient of determination of 0.98. Fruit classification based on the estimated minimum diameter of the equatorial section achieves a low success rate of 84.6%, which is significantly improved using a linear combination of the length/maximum diameter of the equatorial section and projected area/length ratios, reaching 98.3%. Thus, it is possible for Chinese kiwifruit sorting lines to reach international standards of grading kiwifruit on fruit shape classification by adding a single camera.

## 1. Introduction

Kiwifruit is a subtropical fruit that belongs to the family Actinidiaceae and has rapidly spread from China to other parts of the world due to its easy adaptability to local climatic conditions [1]. It is considered as one of the best fruits due to its high nutritive values rich in vitamin C and other mineral elements [2]. China is the largest country of the world for cultivating kiwifruits, where the province of Shaanxi provides the largest contribution with about 70% and 33% of the domestic and global productions [3], respectively. In 2011, the cultivation area of kiwifruits in Shaanxi was around 47,258 hm^2^, while the total production reached 735,748 t, which made Shaanxi the largest kiwifruit plantation area in the world [4]. However, only 2134 t was exported at US$125.97/t in 2010, which accounted for only 0.2% of the year’s production. Conversely, 62,990 t was imported at US$171.89/t in the same year [5], which is probably caused by poor management and grading technology in China kiwifruit industry.

Fruit shape is one of the most important quality parameters for evaluation by customer preference [6,7]. Many studies have been conducted on assessment of fruit shape and these have been used to grade fruits with the help of machine vision, such as apple [8,9,10], tomato [11,12], potato [13,14], and strawberry [15]. Venkatesh et al. [9] used a single camera to capture five different views of an apple and classified it into spherical, ellipsoid, or raboloid shapes by calculating eccentricity and ratio of polar diameter and equatorial diameter. Zhang and Wu [10] proposed eight shape features (area, perimeter, euler, convex, solidity, minor length, major length, and eccentricity) with color histograms and texture features to classify apple and other 17 fruits using a multi-class kernel support vector machine method, and reached a success rate of 88.2%. Arjenaki et al. [11] used one camera to acquire tomato images and developed an image processing algorithm to sort tomatoes into oblong or circular shape based on eccentricity. Elmasry et al. [13] extracted perimeter, centroid, area, moment of inertia, length and width from image to describe potato which being classified into regular and irregular shapes and reached a classification rate of 96.2%. For kiwifruit, Rashidi and Seyfi [16] proposed a classification of fruit shape by applying the analysis of outer dimensions based on length/width and width/thickness ratios, and found that they are effective for determining normal and misshapen fruit.

Fruit shape has been included by many international and national standards of kiwifruit grading as a minimum requirement using the word “well formed” [17,18]. The width/thickness ratio is also used in many kiwifruit grading standards to assess fruit shape besides the minimum requirements of well-formed fruit. Concerning the marketing and commercial quality control of kiwifruit, for example, the United Nations Economic Commission for Europe (UNECE) standard FFV-46 [18] and East African standard CD/K/013:2010 [17] classify fruits into three grades based mainly on fruit weight and ratio of minimum diameter of the equatorial section (MiDES, same as thickness in other studies) to maximum diameter of the equatorial section (MaDES, same as width in other studies) in addition to minimum and maturity requirements.

However, most kiwifruit grading standards in China are applied to sort kiwifruit in different grades using fruit weight without the fruit shape. For example, NY/T 1794-2009 standard [19] classifies kiwifruits into three grades according to their skin damage and weight, and DB440300/T 25.7-2006 standard [20] divides kiwifruits into three grades only by their weight [21]. Consequently, Chinese fruit companies rely on those standards to grade kiwifruits and send them to the domestic market [22]. This poor grading system in China results in low exportation and shares in international kiwifruit markets. It is urgent to improve the sorting lines of kiwifruits in China using fruit shape based on the international standard so as to expand the exportation.

Most studies on classifying fruit grades based on fruit shape are mainly using multi-cameras to capture fruit images from different angles or using a single camera to capture multi-views of a fruit. However, it is costly using multi-cameras and difficult to rotate a fruit with the exactly required location in real sorting lines. It is practical to use a single camera above the fruit in a fixed distance to take fruit shape images on a conveyor belt and then to easily determine the fruit length, MaDES, and projected area (PA) using machine vision. It is desirable to apply the new grading technology based on fruit shape from one camera into the current China kiwifruit sorting lines equipped with weight sensors for achieving international grading levels.

The objectives of this study are to (1) estimate the MiDES from the weight and the measurable parameters using machine vision with one single camera; (2) classify kiwifruit based on the weight and ratio of estimated MiDES and MaDES according to the international standard (FFV-46); and (3) predict and validate fruit volumes to ensure the fruit grading accuracy using practical sample measurements.

## 2. Materials and Methods

### 2.1. Sample Preparation and Image Acquisition

The most popular and common kiwifruit cultivar “Hayward” in Shaanxi is studied in three consecutive years from 2013 to 2015. Sound and well-formed fruits without damage and abnormal external moisture are selected from the Meixian Kiwifruit Experimental Station (34°07′39″ N, 107°59′50″ E, and 648 m in altitude) of the Northwest A&F University with a total number of 150, 160, and 180 in 2013, 2014, and 2015, respectively. The 490 “Hayward” kiwifruit samples used in this study are randomly divided into two groups: the first group is used to develop the calibration models (140 samples, 40 from 2013, 45 from 2014, and 55 from 2015) and another for predicting MiDES and volume, and model validation (350 samples).

**Figure 1:**
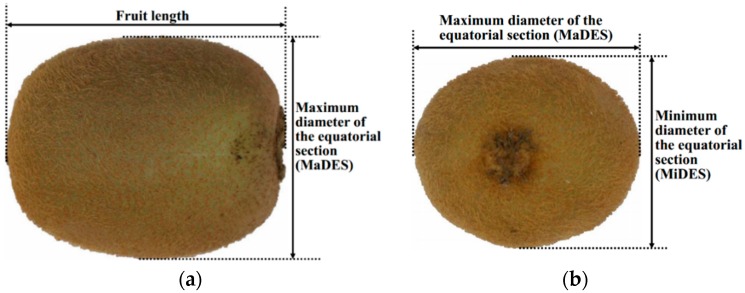
Definitions of fruit length, maximum diameter of the equatorial section (MaDES), and minimum diameter of the equatorial section (MiDES). (**a**) Top view of a kiwifruit; (**b**) Side view of the kiwifruit.

Each sample is firstly placed on a coordinate paper until it has no more rolling and then is positioned in the center of the target field by view of a digital camera (Canon EOS 600D, Canon Inc., Tokyo, Japan). The sample is then captured to a red-green-blue (RGB) color image by the camera with fixed aperture of F8.0 and shutter speed of 1/125 s where a vertical distance between the paper and the camera is set to be 30 cm. After that, its weight is measured by a digital balance (PTT-A1000, HuaZhi Scientific Instrument Co., Ltd., Fuzhou, China) with a sensitivity of 0.01 g. Next, sizes including length, MaDES, and MiDES (as shown in Figure 1) are measured by a Vernier caliper (K15G078575, Guilin Guanglu Measuring Instrument Co., Ltd., Guilin, China) with a resolution of 0.1 mm. Finally, the fruit volume is gauged by the water displacement method (WDM). All the measurements are replicated three times.

### 2.2. Image Pre-Processing and Segmentation

**Figure 2:**
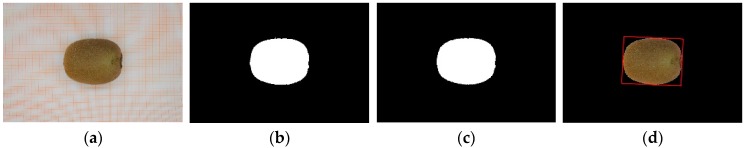
Steps of measuring kiwifruit sizes by image processing methods. (**a**) Original red-green-blue (RGB) color image in a coordinate paper; (**b**) black and white image after using the threshold technique; (**c**) fruit projected area image by eliminating noises using an area threshold method; and (**d**) computed minimal bounding rectangle (red box) of the fruit area to calculate fruit length and MaDES.

Image processing method is used to analyze the fruit images. The RGB color image of each kiwifruit (Figure 2a) is firstly converted to a grayscale one. It is transformed by the National Television Standard Committee standard, which is based on an optimal human perception [23]. The conversion formula is given as follows:

gray = 0.299 × red + 0.587 × green + 0.114 × blue(1)

where gray represents 256 different shades of gray tone from black (0) to white (255), red, green, and blue are the three primary colors of RGB space which also range from 0 to 255.

Then, the threshold technique is used to select a region of interest on the grayscale image and then convert it to a black and white image with pixel values of 0 or 255. From the grayscale image, pixel values less than a threshold are converted to 0 (black) and those higher than the threshold are converted to 255 (white), producing a black-and-white image for each kiwifruit (Figure 2b). The threshold value is determined using the Otsu method [24], which chooses the threshold to minimize the intraclass variance of the black and white pixels. Next, an area threshold method is employed to eliminate the small area noises, as shown in Figure 2c [25]. This method is based on finding the biggest area of neighboring white pixels in the image and eliminating all areas, which are smaller than 1/20 of the biggest area. The resulting binary image is then used to calculate the sizes of the kiwifruits for achieving their shapes.

### 2.3. Extracting and Calculating Shape Parameters

The fruit length, MaDES, and PA are directly determined by image processing method. However, the MiDES is difficult to be measured automatically by the image because the side with the MiDES would be on the conveyor belt during grading on the sorting line caused by the special shape of “Hayward” kiwifruit and its free rolling. Therefore, the MiDES is predicted using the above measurable parameters (weight, length, MaDES, and PA).

After segmentation, a minimal bounding rectangle method (Figure 2d) is applied to calculate the fruit length and MaDES by finding the minimum and maximum values in the horizontal and vertical axes of white area [26]. Then, pixel number of length (PL) and width (PW) of the minimal bounding rectangle is used to calculate the fruit length (L, mm) and MaDES (mm). The pixel number of projected area (PPA) of the fruit is obtained by counting the number of white pixels in the binary image (Figure 2c) and employed to estimate the fruit projected area (PA, mm^2^). Finally, the real sizes measured by image processing method are determined by the ratio (RA) of 1 mm^2^ to pixels counted from the number of pixels in the smallest square of 1 mm × 1 mm on the coordinate paper as follows:

L = PL/RA^1/2^ (2)

MaDES = PW/RA^1/2^ (3)

PA = PPA/RA(4)

The measurable fruit sizes based on images are compared with the manually measured ones. The known weight is used to estimate the MiDES in the calibration dataset by a stepwise multiple linear regression (SMLR) method. All the measurable parameters (weight, length, MaDES, and PA) of fruit samples are analyzed to identify the significant parameters and establish a linear relationship of the MiDES with the selected parameters.

### 2.4. Class Definition

The international standard FFV-46 [18] is employed in this study to grade kiwifruits into different classes. Besides the minimum and maturity requirements, the fruit is graded in three classes according to the fruit shape and weight. “Extra” class: the MiDES to MaDES ratio (MMR) must be 0.8 or greater, and minimum weight is 90 g; class I: the MMR must be 0.7 or greater, and minimum weight is 70 g; and class II: minimum weight is 65 g; The remaining samples are named the “Reject” class in this study. The kiwifruit samples are pre-classified (actual classes) based on the above classification method and the results of grading (estimated classes) are analyzed based on the estimated MiDES.

### 2.5. Fruit Volume Estimation

Although fruit volume is not mentioned in the international standards, it is very important and helpful in management for packaging, transportation and marketing operations [27,28]. If the volume and the weight (determined by a mechanical weighing device on the packing line) of the fruit are known, it would be easy to compute the fruit density, which is a useful measure for detecting frost damage or to estimate dry matter or soluble solids [29]. However, it is difficult to measure volume by common methods of gas displacement and WDM automatically, especially in a sorting line [30].

Rashidi and Gholami [31] determined kiwifruit volume using ellipsoid approximation and image-processing methods by placing a digital camera above the fruit and captured two images before and after manually rotating the fruit by 90° around its longitudinal axis with satisfied accuracy as compared to the ellipsoid approximation method. The criterion they proposed for this volume estimation method should select samples with MMRs lower than 0.85, which is also considered in this study. In addition, it is difficult or costly to rotate a fruit by 90° around its longitudinal axis in the sorting line. Therefore, the kiwifruit volume is estimated without fruit rotation by analyzing the weight and measurable shape parameters based on images. The SMLR methods are employed again to analyze those measurable parameters (weight, length, MaDES, and PA) of fruit samples in the calibration dataset to identify significant parameters and establish the estimation model for volume.

## 3. Results and Discussion

### 3.1. Typical Physical Properties

The average weight, length, MaDES, MiDES, and volume of all 490 samples are found to be 97.9 g, 65.9 mm, 54.1 mm, 46.2 mm, and 93.9 cm^3^, respectively (Table 1). They are close to those of ‘Hayward’ kiwifruit from the same area with the values of 96.9 g, 66.6 mm, 52.1 mm, 46.5 mm, and 96.3 cm^3^, respectively [32]. The similar results are also obtained by Razavi and BahramParvar [1] for Iranian “Hayward” kiwifruit to be 98.7 g, 68.0 mm, 50.3 mm, 46.4 mm, and 102 cm^3^, respectively. MaDES/length ratio (MLR) is 82.1%, which is higher than the reported value (79.8%) by Razavi and BahramParvar [1], but close to that (81.0%) obtained by Lorestani and Tabatabaeefar [33].

The “Extra” class is significantly heavier, longer, wider, thicker, and bigger than the other three classes, whose average weight, length, MaDES, MiDES, PA, and volume are found to be 113.0 g, 70.9 mm, 56.9 mm, 49.9 mm, 37.8 cm^2^, and 108.7 cm^3^, respectively (Table 1). For classes I and II, they are different in the length (*p* < 0.05), but not in the other physical properties (*p* > 0.05). This is probably caused by 31.3% fruit samples of class I supposed to be classified into higher class based on the weight, but actually being classified into class I because of their low MMR values. It is the same for 20% fruit samples of class II. Those samples are flattened in shape and have high MaDES values than the others in the same classes, which induce higher average MLRs (82.4% and 86.1%) but lower MMRs (84.9% and 85.2%) in the classes I and II, respectively. Especially in class II, some flattened samples have weights over 90 g but with MMRs less than 0.7, resulting in the highest standard deviations (SDs) of weight, MaDES, PA, volume, MLR, and MMR among all the classes (Table 1). However, the SDs of length (4.6 mm) and MiDES (3.1 mm) for the Class II samples are lower than those (7.5 mm and 5.8 mm) of the total samples, respectively. The results also indicate that the length and MiDES of the flattened samples are not different from those of the others in the Class II. It is the same for 31.3% fruit samples of class I that have weights over 90 g but with MMRs from 0.7 to 0.8. Fruit samples classified into the “Reject” class are due to their weight less than 65 g.

### 3.2. Fruit Sizes Measured by Image Processing Method

**Figure 3:**
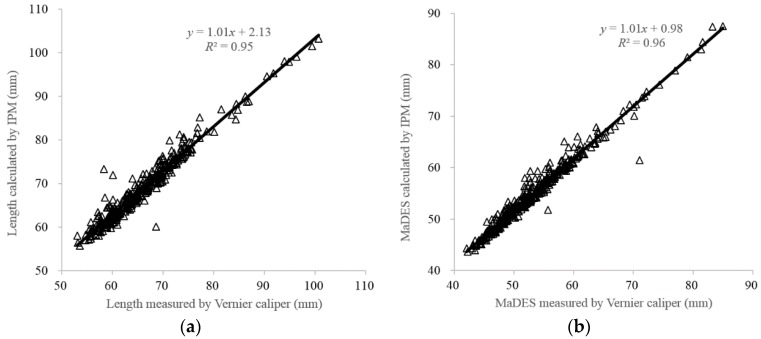
Comparison of length (**a**) and MaDES (**b**) between calculation by image processing method and measurement by a Vernier caliper.

Figure 3 shows a comparison of length and MaDES between calculation by image processing method and measurement by Veriner caliper. A good result of length and MaDES using image processing method is obtained since higher coefficients of determination (*R*^2^) values (0.95 and 0.96, respectively) are achieved. But fruit sizes measured by image processing method are systematically larger than those obtained using a Vernier caliper, since the positive intersection and slope values are obtained in the correlations (Figure 3). The possible reason for larger fruit sizes measured by the image processing method is that the surface hair is recognized as fruit area in the minimal bounding rectangle method [26]. The average surface hair length of “Hayward” kiwifruit is about 0.51 mm [34], which is nearly half of the positive intersection value (0.98 mm) for the correlation of MaDES (Figure 3b). This systematic error could thus be reduced by subtracting this value. For length, some samples have slightly dropped shoulders or calyces very slightly below the shoulders might result in larger values based on the minimal bounding rectangle method in the image processing method as compared to the manual measurement. This is also probably caused by pushing casually the external jaws on to the fruit surface using a Vernier caliper. This result is in good agreement with that of Chalidabhongse et al. [35] who reported that mango lengths and widths measured using the image processing method are higher than those obtained manually using a Vernier caliper. Thus, the machine vision-based measurement method could be reliable and accurate for measuring the fruit size with high speed and convenience.

In addition, as the sizes of fruit increase, the image processing method overestimates the kiwifruit sizes, which is consistent with the results of watermelon size estimation [28]. This is because of the change in distance between the camera and the fruit surface. Although the distance between the camera and the holder is constant, the distance between the fruit surface and the camera is reduced with increasing fruit size. However, this effect is much smaller in kiwifruit as compared to watermelon. The negative effects on the image processing method could be possibly avoided.

### 3.3. MiDES Estimation

Statistical results of the SMLR method for determining the MiDES are presented in Table 2. It indicates that the weight, length, and MaDES are the most crucial parameters in determining MiDES value since they all have corresponding *p* = 0.00 with significance. While the PA had a *p* = 0.49 without significance and the smallest *t* of −0.69. A model for estimating MiDES by the fruit weight, length, and MaDES is thus obtained. The F value of this model is 307.46, and the corresponding *p* value (0.00) is <0.05, showing that the linear relation between the estimated MiDES and selected parameters is significant and the established model is satisfied.

The results show that the estimated MiDES obtained by the model is 46.1 mm on average with a standard deviation of 5.2 mm for all samples (Table 1), which are almost the same as those manually measured values for each class. The small difference indicates the acceptable accuracy of the estimation model.

**Figure 4:**
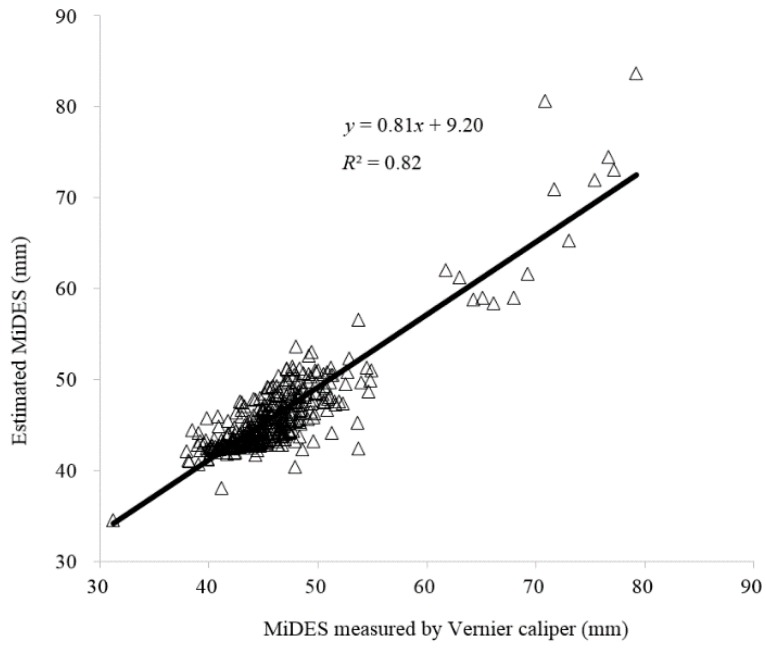
Comparison of MiDES between estimation and measurement by a Vernier caliper in the validation dataset.

However, the smaller *R*^2^ value (0.82) is obtained in the validation dataset for MiDES correlations between estimation and measurement with the positive large intersection (9.20 mm) and small slope value (0.81) as shown in Figure 4. The estimated MiDES value is systematically larger than the measured one below 48 mm, but over 48 mm, this trend is reversed. The small MiDES difference between estimation and measurement for all-class samples suggested that the linear model is acceptable. However, nonlinear models might be applied in future research to further improve the estimation accuracy.

### 3.4. Fruit Classification

Comparison results of six ratios based on the four measurable parameters for groups of actual classes in the estimated ones are listed in Table 3 according to the validation dataset of 350 samples. The low *R*^2^ between the estimated MiDES and the measured values (Figure 4), 41 (11.7% of all classes) samples are thus wrongly classified into the classes above their actual ones. Among them, 28 samples (24.8% of class I) from class I are classified into the “Extra” class and 13 samples from class II (13.7% of class II) are classified into class I, while 13 (3.7% of all classes and 11.2% of “Extra” class) samples from the “Extra” class are wrongly classified into class I. The “Reject” class samples are all correctly classified since their weight is less than 65 g. In all, 84.6% samples are correctly classified based on the estimated MiDES.

To improve the classification rate, the discrimination between correctly and wrongly classified samples is analyzed based on ratios of the measurable parameters, such as weight, length, MaDES, and PA. As shown in Table 3, the length/MaDES and PA/length ratios are significantly different between the two groups of samples from the actual “Extra” class and the actual class I in the estimated “Extra” class, and among the three groups of samples from the actual “Extra” class, class I and class II in the estimated class I. In addition, the weight/length, weight/MaDES, and MaDES/PA ratios (except weight/PA ratio) are found to be significantly different between the two groups of wrongly classified and correctly classified samples in the estimated class I, respectively.

**Figure 5:**
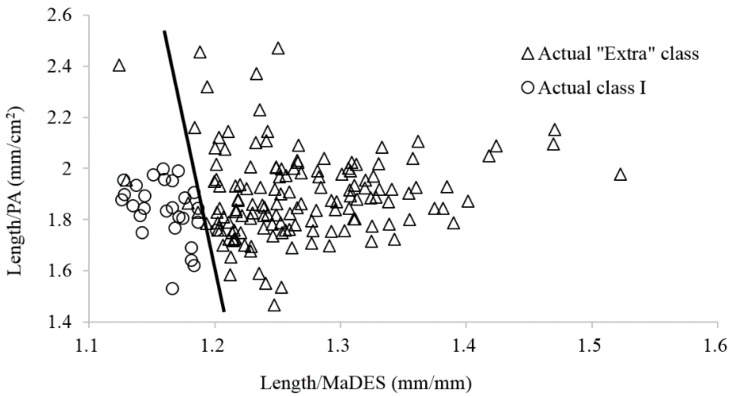
Improved classifications of samples in the estimated “Extra” class based on a linear correlation between the length/MaDES and length/PA values.

**Figure 6:**
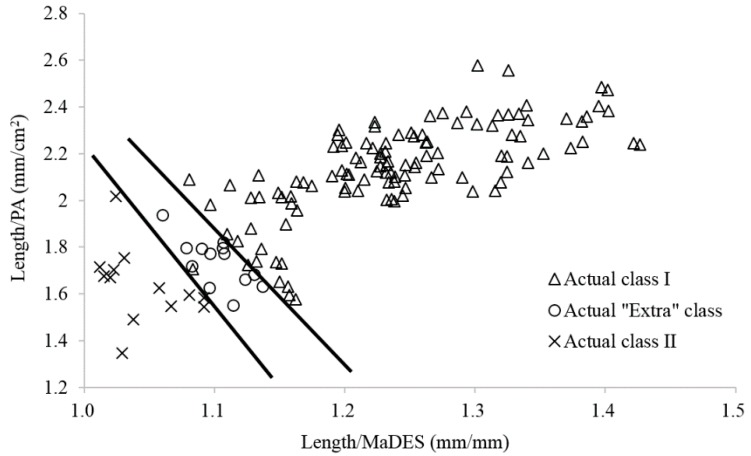
Improved classifications of samples in the estimated class I based on two linear combinations of length/MaDES and length/PA values.

Therefore, the length/MaDES and length/PA ratios are selected to improve the classification rate in the estimated “Extra” class and class I. As shown in Figure 5, a linear combination of the length/MaDES and length/PA ratios could separate the actual class I from the actual “Extra” class in the estimated “Extra” class. Thirty-one samples are grouped into class I. Among them, four samples are from the actual “Extra class”. While for the estimated class I, as shown in Figure 6, all of the 13 samples of the actual class II are separated from the other two classes, and all of the 13 samples of actual “Extra” class are also separated from the other two classes with two linear combinations of the length/MaDES and length/PA ratios, although two samples of actual class I are grouped into the actual “Extra” class. In all, six samples are wrongly classified, and the classification rate is thus improved to 98.3%.

### 3.5. Volume Estimation

Statistical results of the SMLR method for determining the volume are presented in Table 4. It indicates that the weight and length are the most important parameters in determining volume value since they both have corresponding *p* < 0.05 with significance, while the PA and MaDES have *p* values of 0.83 and 0.62 without significances, respectively. A linear model for estimating volume by the fruit weight and length is thus obtained. The *F* value of this model is 1.82 × 10^3^, and the corresponding *p* value (0.00) is less than 0.05, showing that the linear relation between the estimated volume and selected parameters is significant and the established model is valid and reliable. The estimated volume is 0.21% larger than the measured one by WDM in average for all samples, and the mean absolute difference between them is 2.5% (2.3 cm^3^) with the SD of 2.2% (2.0 cm^3^), as shown in Table 1. This is smaller than the results on kiwifruit with the difference of 8.1% (6.1 cm^3^) and the SD of 3.1% (2.9 cm^3^) using the ellipsoid approximation method reported by Rashidi and Gholami [31].

**Figure 7:**
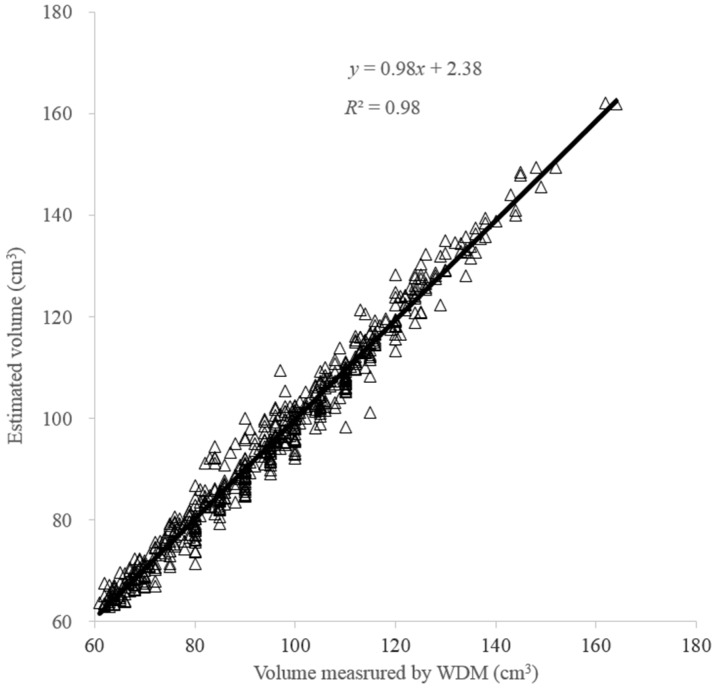
Comparison of fruit volume between estimation and measurement by water displacement method (WDM) in the validation dataset.

Figure 7 shows a good linear relationship of the fruit volume between estimation and measurement by WDM with a high *R*^2^ of 0.98 from the validation dataset. The small intersection (2.38 cm^3^) and the slope (0.98) close to one also indicate a good agreement between estimated and measured volume with small systematic error.

This good estimation result could be achieved using the uniform and consistent samples selected from the Meixian Kiwifruit Experimental Station of the Northwest A&F University, where a professional and standard production was provided. The effective results need to be confirmed with the representative samples from different producers and areas.

In addition, the currently grading classes defined by the China kiwifruit industry are mostly determined by fruit mass using the low-accuracy weight sensor. As proposed by this study, the image processing methods based on fruit shape could be acceptably applied with a combination of the weight sensor in modern post-harvest handling processes. However, the kiwifruit mass estimation should also be studied based on image processing methods to further improve the throughput and classification rate. Several approaches for mass estimation using image processing methods have been developed for various fruits, e.g., apples [36,37], citrus fruits [26,38,39], lemons [9], and mangoes [40,41]. These successful mass estimation methods have potential for image processing methods to provide the kiwifruit industry and consumers with an effective and reliable classification method.

## 4. Conclusions

Fruit shape is an important quality parameter for customers and included in many international and national standards for kiwifruit grading, but not in the China kiwifruit industry. To demonstrate the feasibility for classifying kiwifruit into shape grades by adding a single camera to current Chinese sorting lines equipped with weight sensors, the image processing method is employed to calculate fruit length, MaDES, and PA. A stepwise multiple linear regression method is applied to select significant variables for predicting MiDES and volume and to establish corresponding estimation models. The fruit length and MaDES calculated by the image processing method reach high *R*^2^ (0.95 and 0.96, respectively) to that measured by a Vernier caliper. The length, MaDES, and weight are selected to predict the MiDES by the SMLR method. *R*^2^ of 0.82 is obtained for the correlation between estimated MiDES and the manually measured one, resulting in a low success rate of 84.6%. The classification rate is largely improved using the linear combination of the length/MaDES and PA/length ratios and reached 98.3%. In addition, the weights and lengths are selected for estimating the fruit volume, which is in good agreement (*R*^2^ = 0.98) with the measured one. The machine vision technology holds the potential for the Chinese kiwifruit sorting lines to reach the international standards of grading kiwifruit based on fruit shape classification by adding a single camera with fruits resting on their flat face. Further research needs to be conducted on developing integrated machine vision systems for fruit quality control and uniform packaging according to color, weight, and shape.

## Figures and Tables

**Table 1:** Typical physical properties and estimated parameters (Mean ±standard deviation) of all kiwifruit samples in different classes.

Classes	No.	W (g)	L (mm)	MaDES (mm)	MiDES (mm)	MiDES_est_ (mm)	PA(cm^2^)	V (cm^3^)	V_est_ (cm^3^)	MLR (%)	MMR (%)
“Extra” class	163	113.0 ± 15.0 ^a,^*	70.9 ± 7.5 ^a^	56.9 ± 6.6 ^a^	49.9 ± 7.0 ^a^	49.0 ± 6.5 ^a^	37.8 ± 4.4 ^a^	108.7 ± 14.9 ^a^	108.8 ± 14.4 ^a^	80.3 ± 4.9 ^a^	87.7 ± 5.2 ^a^
Class I	157	90.7 ± 19.0 ^b^	63.6 ± 4.7 ^b^	52.4 ± 5.3 ^b^	44.1 ± 2.2 ^b^	44.7 ± 2.4 ^b^	31.7 ± 5.8 ^b^	86.5 ± 18.5 ^b^	87.4 ± 18.1 ^b^	82.4 ± 6.1 ^a,b^	84.9 ± 7.8 ^a^
Class II	133	80.8 ± 28.2 ^b^	59.4 ± 4.6 ^c^	51.4 ± 10.6 ^b^	42.5 ± 3.1 ^b^	42.9 ± 2.2 ^b^	29.1 ± 9.1 ^b^	77.5 ± 27.1 ^b^	77.8 ± 26.6 ^b^	86.1 ± 13.7 ^b^	85.2 ± 13.5 ^a^
“Reject” class	37	64.0 ± 0.7 ^c^	58.8 ± 2.6 ^c^	47.1 ± 3.3 ^c^	41.4 ± 3.4 ^c^	41.5 ± 0.7 ^c^	24.3 ± 0.7 ^c^	61.8 ± 2.4 ^c^	62.1 ± 0.7 ^c^	80.4 ± 5.9 ^a^	87.9 ± 5.5 ^a^
Total	490	97.9 ± 23.6	65.9 ± 7.5	54.1 ± 7.5	46.2 ± 5.8	46.1 ± 5.2	33.7 ± 7.0	93.9 ± 22.9	94.1 ± 22.6	82.1 ± 7.7	86.2 ± 8.2

W—weight; L—length; MaDES—maximum diameter of the equatorial section; MiDES—minimum diameter of the equatorial section; MiDESest—estimated MiDES; PA—projected area; V—Volume; MLR = MaDES/length; MMR = MiDES/MaDES. * Different letters within a column indicate that means are significantly different (Duncan’s test, *p* < 0.05) among four classes. * Different letters within a column indicate that means are significantly different (Duncan’s test, *p* < 0.05) among four classes.

**Table 2:** Statistical results of the stepwise multiple linear regression (SMLR) model for determining MiDES of the kiwifruits.

Parameters	Coefficients	*t*	Significance
Weight (g)	−0.08	−6.36	0.00
PA (cm^2^)	−3.71 × 10^−4^	−0.69	0.49
Length (mm)	0.66	18.72	0.00
MaDES (mm)	0.24	6.02	0.00
Constant	−2.36	-	-

**Table 3:** Comparison of six ratios (Mean ± standard deviation) based on the four measurable parameters for groups of actual classes in the estimated ones from the validation dataset (350 samples).

Estimated Class	“Extra” Class		Class I			Class II	“Reject” Class
Actual Class	“Extra” Class	Class I	“Extra” Class	Class I	Class II	Class II	“Reject” Class
Fruit samples	103	28	13	85	13	82	26
Weight/Length	1.58 ± 0.15 ^a,^*	1.62 ± 0.13 ^a^	1.74 ± 0.13 ^a,^*	1.37 ± 0.19 ^b^	1.81 ± 0.17 ^a^	1.29 ± 0.36	1.09 ± 0.04
Weight/PA	2.98 ± 0.15 ^a^	2.92 ± 0.11 ^a^	3.02 ± 0.16 ^a^	2.84 ± 0.10 ^a^	2.86 ± 0.15 ^a^	2.75 ± 0.12	2.64 ± 0.08
Weight/MaDES	1.99 ± 0.21 ^a^	1.94 ± 0.18 ^a^	1.95 ± 0.18 ^a^	1.67 ± 0.17 ^b^	1.84 ± 0.20 ^a^	1.51 ± 0.17	1.36 ± 0.09
Length/MaDES	1.26 ± 0.05 ^a^	1.18 ± 0.04 ^b^	1.12 ± 0.03 ^a^	1.23 ± 0.07 ^b^	1.02 ± 0.03 ^c^	1.20 ± 0.15	1.25 ± 0.10
Length/PA	1.89 ± 0.17 ^a^	1.81 ± 0.11 ^b^	1.74 ± 0.05 ^a^	2.10 ± 0.21 ^b^	1.58 ± 0.11 ^c^	2.22 ± 0.34	2.42 ± 0.10
MaDES/PA	1.51 ± 0.16 ^a^	1.51 ± 0.10 ^a^	1.56 ± 0.09 ^a^	1.71 ± 0.13 ^b^	1.54 ± 0.14 ^a^	1.84 ± 0.14	1.94 ± 0.12

* Different letters within a row of the same estimated class indicate that means are significantly different (Duncan’s test, *p* < 0.05) between or among actual classes.

**Table 4:** Statistical results of the multiple linear regression (MLR) model for determining volume of the kiwifruits.

Parameters	Coefficients	*t*	Significance
Weight (g)	0.93	134.16	0.00
PA (cm^2^)	5.47 × 10^−5^	0.2147	0.83
Length (mm)	0.09	4.27	0.02
MaDES (mm)	1.65 × 10^−3^	0.46	0.62
Constant	−2.69	-	-

## Abbreviations

The following abbreviations are used in this manuscript:

**Nomenclature**	
MaDES	maximum diameter of the equatorial section of fruit
MiDES	minimum diameter of the equatorial section of fruit
MiDESest	estimated MiDES
MLR	MaDES to length ratio
MMR	MiDES to MaDES ratio
PA	projected area of fruit
PL	pixel number of fruit length
PPA	pixel number of projected area of fruit
PW	pixel number of fruit width
R^2^	coefficient of determination
RA	ratio of 1 mm^2^ to pixels counted from the number of pixels in the smallest square of 1 mm × 1 mm on the coordinate paper
RGB	color intensity values of red, green and blue
SD	standard deviation
SMLR	stepwise multiple linear regression method
WDM	water displacement method for measuring fruit volume
